# Evaluation of Swiss slaughterhouse data for integration in a syndromic surveillance system

**DOI:** 10.1186/1746-6148-10-33

**Published:** 2014-01-31

**Authors:** Flavie Vial, Martin Reist

**Affiliations:** 1 Veterinary Public Health Institute, Vetsuisse Faculty, University of Bern, Liebefeld, Switzerland; 2 Federal Food Safety and Veterinary Office, Liebefeld, Switzerland

**Keywords:** Carcass condemnation, Post-mortem, Meat inspection, Slaughterhouse, Syndromic surveillance, Early detection

## Abstract

**Background:**

We evaluated Swiss slaughterhouse data for integration in a national syndromic surveillance system for the early detection of emerging diseases in production animals. We analysed meat inspection data for cattle, pigs and small ruminants slaughtered between 2007 and 2012 (including emergency slaughters of sick/injured animals); investigating patterns in the number of animals slaughtered and condemned; the reasons invoked for whole carcass condemnations; reporting biases and regional effects.

**Results:**

Whole carcass condemnation rates were fairly uniform (1–2‰) over time and between the different types of production animals. Condemnation rates were much higher and less uniform following emergency slaughters. The number of condemnations peaked in December for both cattle and pigs, a time when individuals of lower quality are sent to slaughter when hay and food are limited and when certain diseases are more prevalent. Each type of production animal was associated with a different profile of condemnation reasons. The most commonly reported one was “severe lesions” for cattle, “abscesses” for pigs and “pronounced weight loss” for small ruminants. These reasons could constitute valuable syndromic indicators as they are unspecific clinical manifestations of a large range of animal diseases (as well as potential indicators of animal welfare). Differences were detected in the rate of carcass condemnation between cantons and between large and small slaughterhouses. A large percentage (>60% for all three animal categories) of slaughterhouses operating never reported a condemnation between 2007 and 2012, a potential indicator of widespread non-reporting bias in our database.

**Conclusions:**

The current system offers simultaneous coverage of cattle, pigs and small ruminants for the whole of Switzerland; and traceability of each condemnation to its farm of origin. The number of condemnations was significantly linked to the number of slaughters, meaning that the former should be always be offset by the later in analyses. Because this denominator is only communicated at the end of the month, condemnations may currently only be monitored on a monthly basis. Coupled with the lack of timeliness (30–60 days delay between condemnation and notification), this limits the use of the data for early-detection.

## Background

Post-mortem meat inspection has the potential to more easily detect diseases (such as bovine tuberculosis [1]) and welfare conditions (such as tail-biting in pigs [2]) which may not be apparent during ante-mortem inspection of the animal upon arrival to the slaughterhouse; and may form the basis for developing strategies that aim to increase production efficiency and animal welfare. The value of meat inspection (as defined by regulation (EC) No 854/2004) as an animal health surveillance tool has been highlighted in several recent reports by the European Food Safety Authority [3-5] even though this value may depend on the disease or welfare condition targeted. Despite this recognition, systematic collection and use of meat inspection data for epidemiological surveillance is scarce at the European Union level [6]. This may stem from the fact that the purpose of meat inspection was historically focused on the detection of zoonotic infections before being recently broadened to encompass the surveillance of animal diseases that pose a lesser risk to public health [7]. Nevertheless, a recent inventory of veterinary syndromic surveillance^a^ initiatives in Europe (Triple-S project) revealed 10 monitoring systems that used (e.g. Sweden) or planned to use (e.g. France) meat inspection data from slaughterhouses [8].

Our study aims to evaluate Swiss slaughterhouse data for integration in a national syndromic surveillance system for the early detection of emerging and re-emerging diseases in production animals. More specifically, we wish to identify gaps and insufficiencies in the current federal meat inspection database and to provide relevant suggestions for its improvement, to allow for future use of Swiss slaughterhouse data for syndromic surveillance purposes. In this context, a syndrome is defined as “a set of non-specific pre-diagnosis medical and other information that may indicate […] a natural disease outbreak” [9]. Whole or partial carcass condemnations following meat inspection could therefore be a valuable syndrome, and indirect indicator of national herd health, to monitor, as seen in Ontario, Canada [10].

### The Swiss meat inspection database

In Switzerland, animals to be slaughtered for meat production need to be taken to an authorised slaughterhouse. Each animal will go through several rounds of inspection: visual inspection ante-mortem, post-mortem carcass inspection and, for some, collection of samples for further testing (e.g. compulsory testing for trichinellosis in pigs). During the post-mortem inspection, carcasses and offal must be examined for alterations of the meat (e.g. colour), signs of particular diseases (in particular notifiable zoonoses), micro-organisms and pathogens, incompletely removed specified risk material (e.g. spinal cord), foreign substances (e.g. medicinal or chemical) and contamination (e.g.: by feces). Depending on the observations made by the meat inspector (none, generalised vs. localised conditions) on the carcass, the carcass can either be 1) classified as entirely fit for human consumption; 2) wholly condemned (this includes organs and blood) or 3) partially condemned (only parts of the carcass unfit for human consumption are removed). Reasons for whole carcass condemnations are listed in the Swiss legislation^b^ and must be reported back to the producers and to the veterinary authorities.

The veterinary services of each Swiss canton are responsible for the meat inspection process in slaughterhouses located in their jurisdiction (Switzerland is made up of 26 cantons, each with their own veterinary authorities). Since 2007, the official veterinarians in charge of meat inspection and the cantonal veterinary services use the FLEKO federal database to communicate the number of animals slaughtered and the meat inspection results to the Federal Food Safety and Veterinary Office (FSVO- the federal body dealing with animal health and welfare issues for the whole Swiss Confederation). Results from meat inspection must be made available to the cantonal services by the meat inspector at least once a month, and the data has to be entered into FLEKO by the cantonal services before the end of the following month. Currently, the FLEKO only contains information about whole carcass condemnations. Partial condemnations do occur at a much higher frequency but their declaration to the FSVO is not compulsory.

We retrospectively describe the meat inspection data routinely collected for cattle, pigs and small ruminants (sheep and goats) slaughtered in Switzerland between 01/01/2007 and 31/12/2012. This is a pre-required first step before attempting to use such data for the real-time detection of temporal aberrations in the number of whole carcass condemnations in a prospective fashion.

## Methods

### Data extraction

This study did not require the approval of an ethical committee. The following data were extracted and derived from the FLEKO database for each slaughterhouse, for each animal category for each month between January 2007 and December 2012: total number of carcasses processed and number of whole carcass condemnations during normal slaughter and emergency slaughter (slaughter of sick or injured animals).

While a specific date is available for each carcass condemnation, the total number of carcasses processed is only calculated at the end of the month, thereby only allowing analyses on a monthly basis. Furthermore, for each whole carcass condemnation, a reason is recorded by the meat inspector using a standardised 4 digit code (no free-text is allowed). At least one main reason (with the options of up to two additional ones) needs to be entered on the meat inspection report.

Condemnation data were available for several animal categories. Up until 2009, cattle were classified according to three age classes (calves (<6 months), young cattle (7–24 months) and adult cattle (>24 months)) and were listed separately in the meat inspection record. From 2010, age classes were simplified to animals younger than six weeks (0.5% of carcasses) or older. However, canton Ticino in Switzerland still retains the old classification system up to this day. To simplify the interpretation, records from all cattle of different ages were analysed together.

The monthly realised price of various non-label meat types at the butchers were obtained from the Swiss Farmer’s Union (Schweizerischer Bauernverband). The monthly prices from the union are calculated based on the weekly prices realised in the slaughterhouses (and weighted according to the number of working days). Prices are in Swiss francs (SFR) per kg of carcass, including VAT. Price of replacement stock for cattle was also obtained from this organisation. Prices are auction prices in SFR for a Brown Swiss cow (most common breed found in Switzerland).

### Mapping

Full addresses for the slaughterhouses were not disclosed, only information on the postcode and the town the slaughterhouses were located was made available. Maps of the cantonal differences in carcass condemnation rates were produced in R using the {sp} package [11].

### Time-series analysis

Seasonality in time series was assessed by using the function “decompose” in R. Time-series (Y[t]) were decomposed into seasonal (S[t]), trend (T[t]) and irregular components (e[t]) using moving averages, following an additive model (Y[t] = T[t] + S[t] + e[t]) or multiplicative model (Y[t] = T[t] * S[t] * e[t]) depending on the homogeneity of the variance over time. The seasonal component extracted from the time-series were observed to identify “peak” month (month in which number of events is highest) and “trough” month (month in which number of events is lowest). Seasonality was removed from the time-series before fitting models investigating the effect of time (trend) and price of commodity.

As the decomposition of the time-series provided strong indications that the trends in the number of events were not linear, generalized additive models (GAM), in the{mgcv} package [12], were used with the variable time as a smoother. The analyses were carried out following these steps:

1) We started by fitting a simple GAM model (assuming independent residuals) and analysing the model residuals, in particular their autocorrelation (acf) and partial-acf plots. These plots informed us whether the correlation of the errors seem to originate from an ARMA (p,q) process (auto-regressive moving average) and which values for p and q may be appropriate.

2) We fitted an ARMA (p,q) model to the GAM residuals and tested whether it provided an adequate model for the correlation structure. If several p and q parameters seemed plausible in step 1, several ARMA (p,q) models were fitted and compared based on AIC and visualisation of the behaviour of ARMA (p,q) model residuals.

3) A second more complex GAM model was then fitted to the original data. The correlation structure of the errors, using the ARMA (p,q) process, was provided as a model input based on steps 1 and 2. Model acceptance was based on behaviour of model residuals.

In each year, slaughterhouses were classified on size based on whether their processing volume was larger or smaller than the median volume calculated over all slaughterhouses for that year. Models were first run on the dataset from all the slaughterhouses before being run separately for larger and smaller slaughterhouses. Reported statistics for the smoother include the estimated degrees of freedom (edf) and result of F- test of whether the smoothed function significantly reduced model deviance. The parametric estimates of the models are listed with t-tests of significance against a null of zero.

## Results & discussion

### Activity of Swiss slaughterhouses

Slaughterhouses are mostly located in the non-alpine region of Switzerland (northern half of the country). The slaughterhouse landscape is very heterogeneous with a few large slaughterhouses dominating the market, while the rest process a low number of carcasses per year (Table 1). This centralisation of the slaughtering business will likely be exacerbated in the future as larger cattle and pig slaughterhouses are receiving increasing number of animals.

**Table 1:** Heterogeneity in the size of Swiss slaughterhouses

	**Pigs**	**Cattle**	**Small ruminants**
Min	1	1	1
Median	194	70.5	87
Max	562,600	155,900	40,510
>100	325	251	250
>1,000	71	25	27
>10,000	14	8	6
>100,000	7	2	NA

Minimum, maximum and median number of carcasses processed in Swiss slaughterhouses in 2012; number of slaughterhouses having processed at least 100/1,000/10,000/100,000 carcasses that same year.

We expected the FLEKO data to be sensitive to the effects of regulatory and economic changes in the industry. The required standards to operate a slaughterhouse in Switzerland changed in November 2005 following the decision by the EU to modify the legislation regulation hygiene standards in facilities processing animal products for countries wishing to export to the EU^b^. Swiss slaughterhouses had until the end of 2008 to adapt their facilities if they did not correspond to the minimum standard set out by the law. This change in the Swiss legislation is probably responsible for the 20% decrease in the number of slaughterhouses between 2007 and 2012 as sub-optimal facilities were being inspected and shut down (Table 2).

**Table 2:** Swiss slaughterhouses and slaughters between 2007 and 2012

	**Pigs**			**Cattle**			**Small ruminants**		
**Year**	**Number of slaughterhouses**	**Number of normal slaughters**	**Number of emergency slaughters**	**Number of slaughterhouses**	**Number of normal slaughters**	**Number of emergency slaughters**	**Number of slaughterhouses**	**Number of normal slaughters**	**Number of emergency slaughters**
2007	673	2,757,363	4,619	743	591,554	11,496	689	273,570	424
2008	648	2,639,138	4,973	718	607,083	11,904	653	276,803	634
2009	611	2,704,967	5,133	686	635,134	10,870	641	260,027	539
2010	597	2,839,485	5,754	667	636,879	10,865	616	270,872	460
2011	571	2,821,459	6,324	637	643,551	10,084	588	272,761	404
2012	534	2,755,187	6,917	602	636,604	10,085	554	259,607	481

Number of slaughterhouses having processed at least one carcass of a particular production animal type in a given year and total number of animals slaughtered per year.

Less than 50% of slaughterhouses were open for 12 months a year. A strong seasonality was observed in the number of slaughterhouses operating with a peak in December (March for cattle) and a trough in July. The December peak reflects the Swiss consumers’ behavior at the time of year and the increased demand for pork meat.

### Traffic through Swiss slaughterhouses

Pigs, cattle and small ruminants constitute 97% of all slaughters recorded in the FLEKO (the rest being made up of rabbits, horses and farmed game). Poultry is a production sector (including slaughter) that is separate from cattle, pigs and small ruminants in Switzerland so that the estimated 55 million birds slaughtered annually (Swiss Farmer Union) are not reported to the FLEKO. Pigs were the most numerous production animals sent to the slaughterhouses between 2007–2012, representing 75% of all normal slaughters declared to the FLEKO. Cattle came second (17%), followed by small ruminants (7%) (Table 2). However, nearly two-thirds of all emergency slaughters were cattle (64%), followed by pigs (33%) with small ruminants only accounting for 3% of emergency slaughters.

We observed a peak in the number of pigs slaughtered in December, and a peak in March for cattle and small ruminants slaughter. A similar pattern was observed for emergency slaughters (Table 3). Cattle production is still seasonal in Switzerland with a peak in calving in late autumn (November). Most calves are conventionally fattened for 3 to 5 months before slaughter, contributing to the March peak in slaughters. Furthermore, a peak in the animals sent to slaughter is to be expected at the end of the winter/early spring when roughage stores have been depleted after the animals had to be kept inside and fed for the whole winter (instead of buying expensive feed). Similarly, cows that have trouble reproducing or producing milk and cows judged unfit to be taken out to alpine pastures during the summer may be preferentially sent for slaughter in the spring. In contrast, there is traditionally a very high demand for pork meat in December and January when more Schüfeli and ham are eaten. While quite a lot of beef is imported in Switzerland, this is less the case for pork meat so that consumer behaviour may strongly influence the number of pigs sent for slaughter. It is also the case with small ruminants, as seen in our “Easter lamb” effect on the number of slaughters in March.

**Table 3:** GAM investigating seasonality, time trend in the time-series and effect of commodity price

		**Peak month**		**Trough month**		**Model**		**Time trend**		**Commodity**	
										**Price effect**	
		**Nb. slaughters**	**Nb. condemn**	**Nb. slaughters**	**Nb. condemn**	**Nb. slaughters**	**Nb. condemn**	**Nb. slaughters**	**Nb. condemn**	**Nb. slaughters**	**Nb. condemn**
Pigs	N	December	December	May	July	ARMA(7,0)	ARMA(0,0)	Yes ↑↓	Yes ↑	Yes	No
	E	January	December	July	July	ARMA(0,0)	ARMA(0,0)	Yes ↑	No	-	-
Cattle	N	March	December	July	January	ARMA(3,2)	ARMA(1,0)	Yes ↑	No	Yes	No
	E	March	March	Nov	Nov	ARMA(3,0)	ARMA(0,0)	Yes ↓	Yes ↑	-	-
Small ruminants	N	March	June	July	February	ARMA(2,1)	ARMA(0,0)	No	No	No	No
	E	March	June	August	November	ARMA(0,0)	ARMA(0,0)	Yes ↑↓	Yes ↑↓	-	-

Residuals were modeled using an ARMA(p,q) process to account for non-independence (when applicable). Results from both normal (N) and emergency (E) slaughters are presented. ↑ denotes a significant increase; ↓ a significant decrease and ↑↓ significant fluctuations.

However, the seasonality pattern observed in the number of carcass condemnations (during both normal and emergency slaughters) was different. We found that the number of condemnations peaked in December for both cattle and pigs. This indicates that the number of carcasses condemned is not solely affected by the total number of carcasses inspected but by other factors too, such as the seasonality in the prevalence of production diseases or in reproduction. This pattern is congruent with the hypothesis that individuals of lower quality are sent to slaughter in winter when hay and food are limited. The winter peak in calving, with its associated recrudescence of dystocia and milk fever cases, may also contribute to this observation. We could not find any satisfying explanations for the peak in small ruminants condemnations in June.

### Pigs

#### *Normal slaughters*

**Figure 1:**
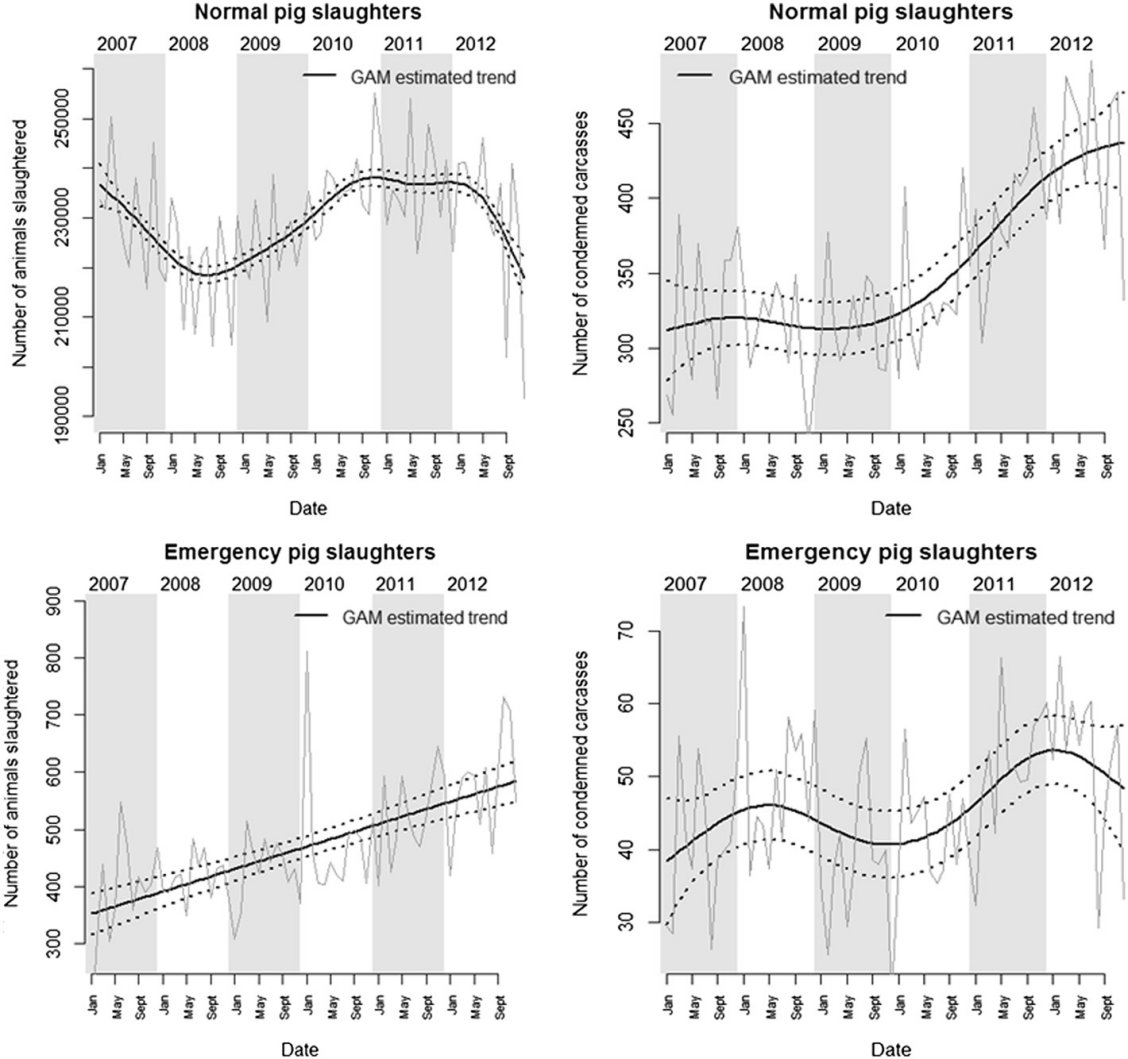
**GAM estimated trends for the number of pig carcasses.** Monthly numbers of pigs slaughtered (left) and condemned carcasses (right) between 2007 and 2012 during normal slaughter (top) and emergency slaughter (bottom) are presented.

We observed a significant increase in the number of pig carcasses being condemned during normal slaughters over the years (EDF = 3.7, F = 11.04, p < 0.001) (Figure 1). This increase was mostly driven by the increase observed in the large slaughterhouses (EDF = 2.7, F = 13.05, p < 0.001) as numbers of unfit pigs in small slaughterhouses did not significantly change (EDF = 1, F = 1.45, p = 0.23). The fluctuating market prices of pig meat (t = -0.13, p = 0.90) did not have a significant impact on the number of pigs being condemned. The total number of carcasses being processed was a significant predictor of the number of unfit carcasses being condemned (t = 2.17, p = 0.03): +0.7 (95% CI 0.1-1.3) for every 1,000 carcasses inspected.

There were significant fluctuations in the number of pigs being slaughtered (EDF = 5.59, F = 13.11, p < 0.001) between 2007 and 2012. These fluctuations were mostly driven by the number of animals being slaughtered in larger slaughterhouses (DF = 8.6, F = 64.6, p < 0.001) as the numbers being processed in smaller slaughterhouses have been decreasing (EDF = 1, F = 22, p < 0.001). The fluctuating market for pig meat had an effect on the number of animals sent for normal slaughter (t = -2.49, p = 0.015): the number of animals slaughtered decreased by around 4,977 (95% CI 1,064- 8,861) for every 1 SFR increase. This counter-intuitive finding may indicate a time-lag between changes in the commodity market and decision-making by farmers to send their animals for slaughters; but should more likely be interpreted as a discrepancy between demand and supply of particular types of meat with a knock-on effect of commodity prices. On the other hand, the numbers of whole carcass condemnations were not significantly linked to market fluctuations even though we may have expected animals of lower quality being sent for slaughter when commodity prices were high. However, it is possible that our monthly data lacks the resolution needed to detect fine-scale “supply and demand” temporal effects on the industry.

#### *Emergency slaughters*

On the other hand, we did not find a significant trend in the number of pigs condemned during emergency slaughter (EDF = 4.4, F = 2.14, p = 0.08). When slaughterhouses were grouped in terms of size, there was no significant trend observed in large slaughterhouses (EDF = 1, F = 0.15, p = 0.70). The significant fluctuations (EDF = 3.97, F = 6.42, p < 0.001) in the number of unfit carcasses being condemned in small slaughterhouses must be interpreted with caution given the small absolute monthly number of condemnations. As for normal slaughter, the total number of emergency slaughters carcasses being processed was a significant predictor of the number of unfit carcasses being condemned (t = 2.37, p = 0.002): +28.7 (95% CI 4.9-52.5) for every 1,000 carcasses inspected.

We also observed an overall increase in the number of emergency slaughters (EDF = 1, F = 53.37, p < 0.001), a trend that was observed in both small (EDF = 1, F = 9.5, p = 0.003) and large (EDF = 1, F = 70.6, p < 0.001) slaughterhouses. It is possible that the unfavorable economic situation for the pig industry in the last 5 years partly explains this trend. There has been an over-production of pigs in Switzerland which has resulted in low prices for pig meat and a campaign urging farmers to try and reduce the number of sows. If farmers are getting rid of less productive (injured or sick) individuals first, we would expect a rise in the number of emergency slaughters in pigs. This may also partly explain the rise in the number of condemnations in pigs sent to normal slaughter that we have observed.

### Cattle

#### *Normal slaughter*

**Figure 2:**
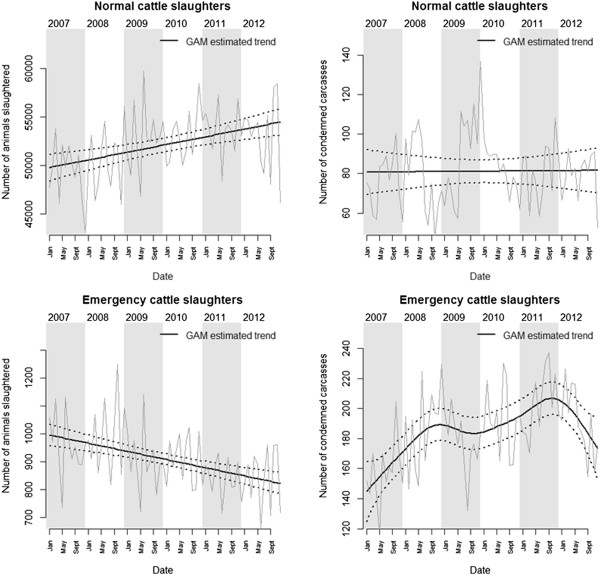
**GAM estimated trends for the number of cattle carcasses.** Monthly numbers of cattle slaughtered (left) and condemned carcasses (right) between 2007 and 2012 during normal slaughter (top) and emergency slaughter (bottom) are presented.

There were no significant fluctuations in the number of cattle being condemned (EDF = 1, F = 0.31, p = 0.60) during normal slaughter (Figure 2). When slaughterhouses were grouped in terms of size, we did not observe significant trends either although there was a tendency for an increasing number of unfit cattle being detected in larger slaughterhouses (EDF = 1, F = 1.4, p = 0.2) and a decreasing number of unfit cattle being detected in smaller slaughterhouses (EDF = 1, F = 1.4, p = 0.2). The total number of carcasses being processed was a significant predictor of the number of unfit carcasses being condemned (t = 3.4, p = 0.001): +1.3 (95% CI 0.6-2.1) for every 1,000 carcasses inspected. The fluctuating market prices for cow (t = 1.23, p = 0.22), heifer (t = 0.01, p = 0.99) and bull (t = 0.02, p = 0.99) meat did not have a significant impact on the number of cattle being condemned, and neither did the price of replacement stock (t = 0.95, p = 0.37).

There was an overall increase in the number of cattle being slaughtered between 2007 and 2012 (EDF = 2.12, F = 7.67, p < 0.001). However, when slaughterhouses were grouped in terms of size, we observed that this increase was driven by the number of animals being slaughtered in larger slaughterhouses (EDF = 4.6, F = 41.4, p < 0.001) as the numbers being processed in smaller slaughterhouses had been decreasing (EDF = 4.6, F = 24.4, p < 0.001). The fluctuating meat and auction market seemed to have an effect on the number of animals sent for normal slaughter. The number of animals slaughtered was lower (t = -3.2, p = 0.002) by around 2072 (95% CI 812–3,333) for every 1 SFR increase in the price of cow meat; was lower (t = -2.94, p = 0.004) by around 1,699 (95% CI 567–2,831) for every 1 SFR increase in the price of heifer meat; and was lower by around 1,755 (95% CI 574–2936) for every 1 SFR increase in the price of bull meat (t = -2.9, p = 0.005). Such relationship between the price of meat and the number of animals slaughtered should not necessarily be interpreted as a causal effect but as a mutual dependence between price and supply. It is thus likely that a decrease in the number of animals sent for slaughter led to an increase in meat price. Furthermore, the number of animals slaughtered was lower (t = -2.9, p = 0.005) by around 300 (95% CI 95–503) for every 100 SFR increase in the price of replacement stock.

#### *Emergency slaughter*

On the other hand, we observed an overall increase in the number of cattle being condemned during emergency slaughters (EDF = 5.2, F = 9.9, p < 0.001), a trend that was driven by the number of condemnations in large slaughterhouses (EDF = 5.7, F = 11.7, p < 0.001). There was no trend in the number of condemnations in small slaughterhouses (EDF = 1, F = 0.52, p = 0.48). As for normal slaughter, the total number of emergency slaughter carcasses being processed was a significant predictor of the number of unfit carcasses being condemned (t = 2.8, p = 0.007): +37.7 (95% CI 11.2-64.2) for every 1000 carcasses inspected. Similarly, we observed an overall decrease in the number of emergency slaughters (EDF = 1, F = 26.76, p < 0.001), a trend that was observed in both small (EDF = 1, F = 16.1, p < 0.001) and large (EDF = 1, F = 26.5, p < 0.001) slaughterhouses.

### Small ruminants

#### *Normal slaughters*

**Figure 3:**
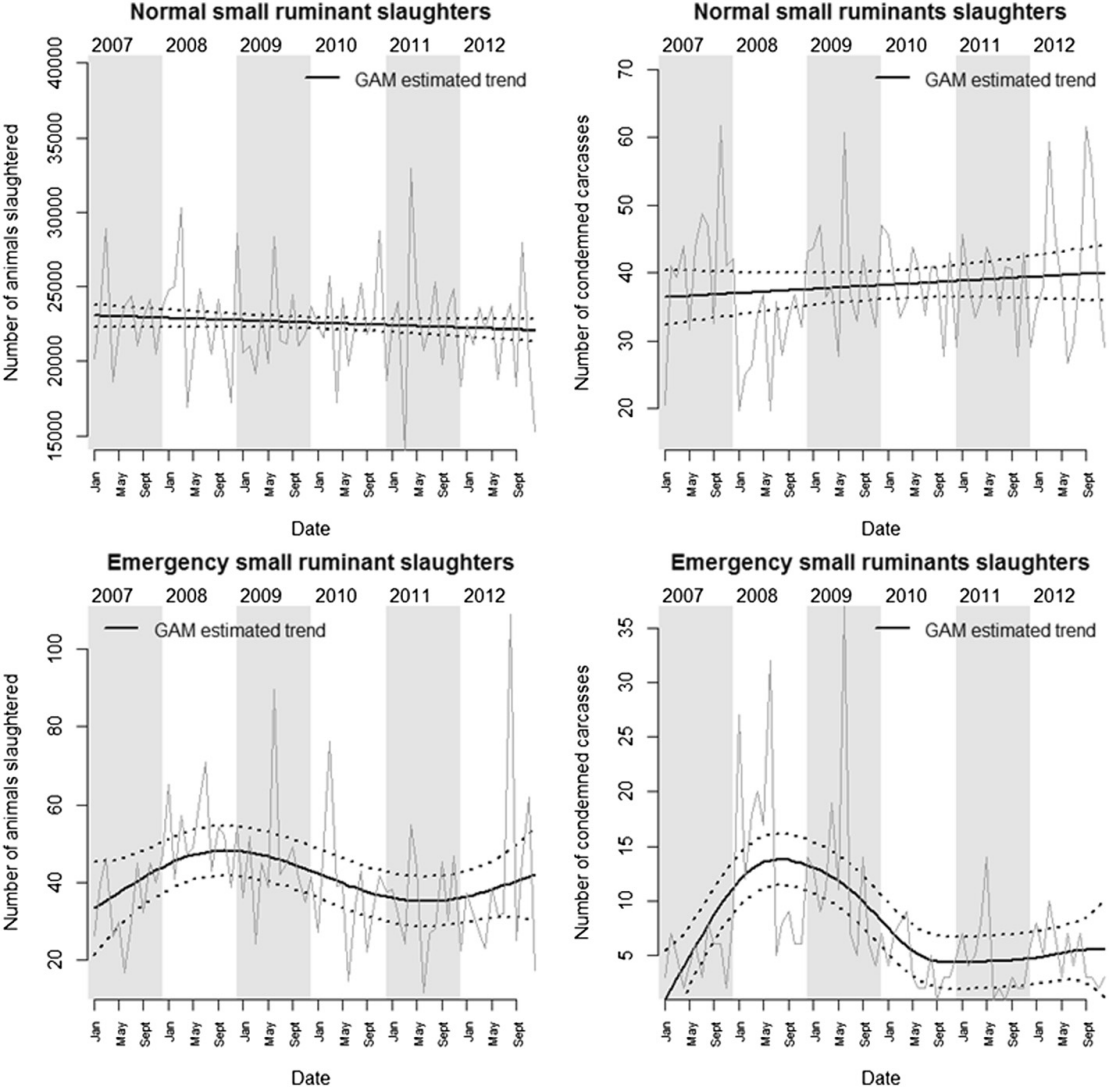
**GAM estimated trends for the number of small ruminant carcasses.** Monthly numbers of small ruminants slaughtered (left) and condemned carcasses (right) between 2007 and 2012 during normal slaughter (top) and emergency slaughter (bottom) are presented.

We did not find significant fluctuations in the number of small ruminants being condemned (EDF = 1, F = 1.06, p = 0.33) during normal slaughter (Figure 3). When slaughterhouses were grouped in terms of size, we did not observe significant trends either although there was a slight tendency for an increasing number of unfit carcasses being detected in large slaughterhouses (EDF = 1, F = 1.02, p = 0.32) but not in small slaughterhouses (EDF = 1, F = 0.003, p = 0.96). The total number of carcasses being processed was not a significant predictor of the number of unfit carcasses being condemned (t = 1.33, p = 0.19) and neither was the price of lamb meat (t = -0.45, p = 0.65).

There were no significant fluctuations in the number of small ruminants being slaughtered (EDF = 1, F = 2.17, p = 0.15). When slaughterhouses were grouped in terms of size, we did not observe significant trends either although there was a tendency for a decreasing number of small ruminants being sent to larger slaughterhouses (EDF = 1, F = 2.7, p = 0.11) and an increasing number of small ruminants being sent to smaller slaughterhouses (EDF = 1, F = 3.31, p = 0.07). The fluctuating market price for lamb meat did not seem to affect the number of animals sent for normal slaughter (t = -0.12, p = 0.91).

#### *Emergency slaughters*

We observed significant fluctuations in the number of emergency slaughters (edf = 3.17, F = 2.93, p = 0.04). However, when slaughterhouses were grouped in terms of size, the time smoother was non-significant for large (EDF = 1, F = 1.25, p = 0.27) and small (EDF = 1, F = 0.41, p = 0.52) slaughterhouses.

We did observe significant fluctuations in the number of unfit small ruminant carcasses detected during emergency slaughter (EDF = 4.8, F = 8.23, p < 0.001). Because of the very low numbers of unfit carcasses detected in small slaughterhouses during emergency slaughter, it was not possible to further explore trends based on slaughterhouse size. The total number of emergency slaughter carcasses being processed was a significant predictor of the number of unfit carcasses being condemned (t = 3.57, p < 0.001): 90.6 (95% CI 40.8-140.4) for every 1,000 carcasses inspected.

### Carcass condemnation rate

The whole carcass condemnation rates following normal slaughters in Switzerland (Table 4) were fairly low and uniform across time and animal type (1–2‰). To put this rate in perspective, whole carcass condemnation rates of 4–8‰ and 3.7‰ for cattle and pigs respectively were reported in Ontario, Canada (Alton et al. [10]; Amezcua et al. [13]); 3.5‰ for pigs in the UK in 2005 [14] and 6.7‰ for cattle in France [3].

**Table 4:** Rates of carcass condemnation per 1,000 carcasses processed per year

**Year**	**Pigs**		**Cattle**		**Small ruminants**	
	**Normal**	**Emergency**	**Normal**	**Emergency**	**Normal**	**Emergency**
2007	1.40	107.53	1.54	167.11	1.81	160.00
2008	1.41	118.69	1.58	193.15	1.34	272.65
2009	1.42	88.28	1.72	200.10	1.85	256.65
2010	1.41	93.49	1.54	214.53	1.68	117.71
2011	1.67	98.03	1.52	244.51	1.66	138.50
2012	1.88	91.56	1.49	227.12	1.91	143.14

On the other hand, condemnation rates were much higher and less uniform following emergency slaughters. The higher rate observed for cattle could be explained by the fact that there are more emergency slaughters in cattle due to accidents on steep alpine pastures in cattle than in pigs, which frequently result in widespread injuries and hematomas. These often lead to the condemnation of the entire carcass at slaughter. Meat inspectors also explained that abscesses on a carcass resulting from an emergency slaughter are more frequently isolated in pigs (resulting in only partial carcass condemnations) than in cattle. Carcass condemnations following emergency slaughters in small ruminants should not be over-interpreted as these involve limited numbers.

#### *Slaughterhouse size and condemnation rates*

We investigated the potential association between slaughterhouse size and condemnation rates in cattle and pig slaughterhouses. We found no such association (χ2 = 0.014, df = 1, p = 0.91) for cattle following normal slaughter. Larger slaughterhouses condemned a higher percentage of cattle carcasses (22.9% versus 5.8%) than small slaughterhouses following emergency slaughter (χ2 = 64, df = 1, p < 0.001). The same phenomenon was observed for large slaughterhouses (9.9% versus 7.4%) condemning pig carcasses following emergency slaughter (χ2 = 18.05, df = 1, p < 0.001); and condemnations of small ruminant carcasses (21% versus 5.8%) following emergency slaughter (χ2 = 49.8, df = 1, p < 0.001). This observation may be the reflect of: 1) meat quality requirements differing between slaughterhouses supplying supermarket operators (often the larger slaughterhouses) and the others; 2) time-constraints: meat inspectors in larger slaughterhouses have less time to dedicate to any particular carcass so that the whole carcass may be condemned even when some parts could have been saved for human consumption; 3) the higher degree of independence of the meat inspector from the producers/industry in large slaughterhouses so that the decisions taken are more often solely based on the observations made during post-mortem meat inspection; 4) animals will often have to travel longer to reach the larger slaughterhouses, increasing the probability of an accident/injury during transport which can result in the animal ending up as an emergency slaughter and being condemned.

#### *Cantonal effect*

**Figure 4:**
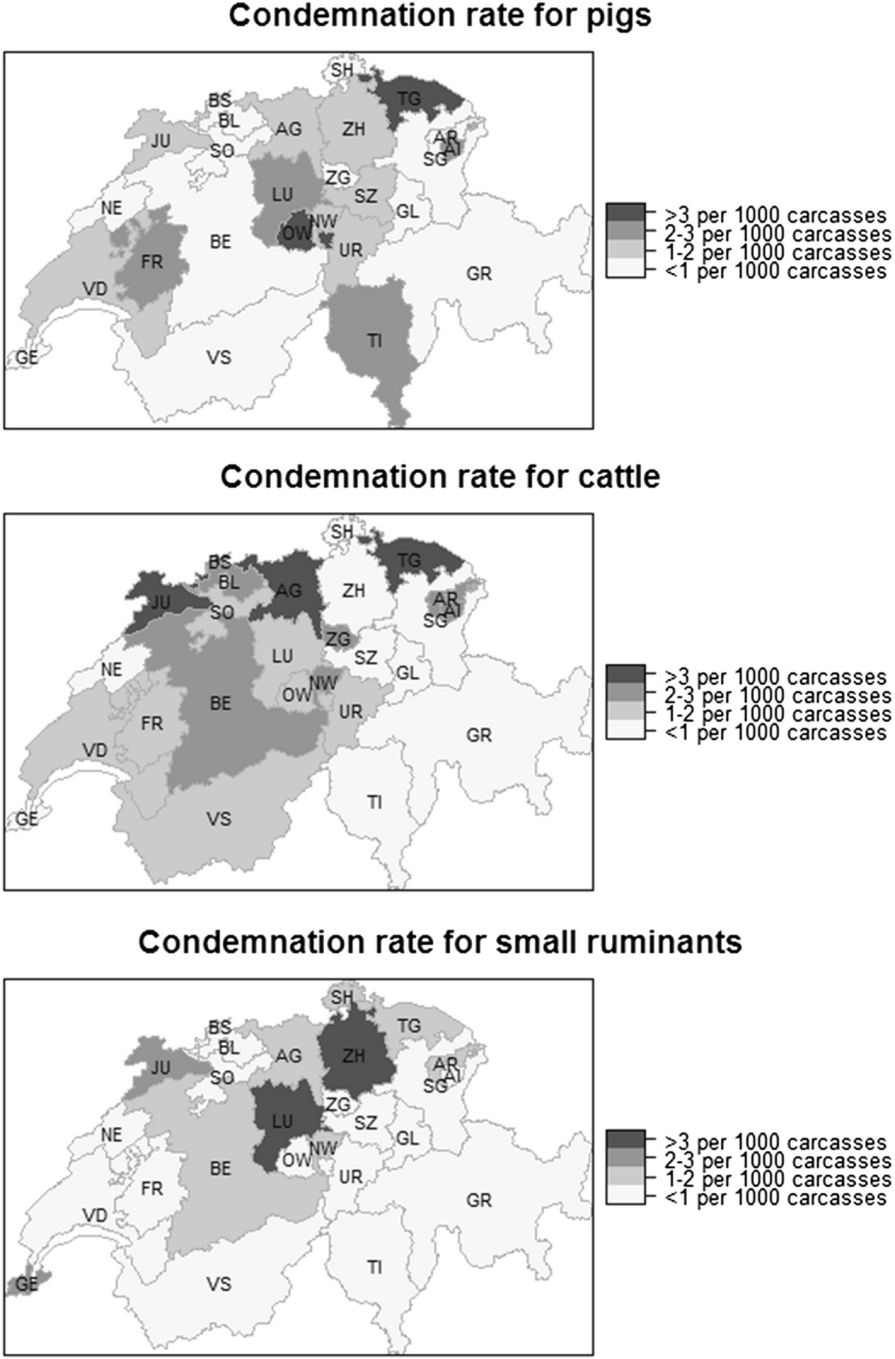
**Cantonal differences in carcass condemnation rate.** Map of Switzerland showing carcass condemnation rate (per 1,000 carcass inspected during normal slaughter) between 2007 and 2012 based on the location of slaughterhouses, and aggregated per canton for pigs, cattle and small ruminants. Swiss cantons: AG Aargau; AI Appenzell I. Rh.; AR Appenzell A. Rh.; BE Bern; BL Basel-Landschaft; BS Basel-Stadt; FR Fribourg; GE Geneva; GL Glarus; GR Graubünden; JU Jura; LU Lucerne; NE Neuchâtel; NW Nidwalden; OW Obwalden; SG St. Gallen; SH Schaffhausen; SO Solothurn; SZ Schwyz; TG Thurgau; TI Ticino; UR Uri; VD Vaud; VS Valais; ZG Zug and ZH Zürich.

Pronounced differences were detected in the rate of carcass condemnation between cantons (Figure 4), possibly echoing the uneven spatial distribution of large slaughterhouses in Switzerland. The data suggest that some slaughterhouses perform a higher percentage of emergency slaughters than others and may “specialise” in processing carcasses of lower quality. Comparing the maps, there appears to be little consistency between the condemnation rates of the cantons for the different type of production animals. Because the differences in condemnation rates observed between countries, and cantons, may not necessarily only reflect differences in herd health but also differences in the population sent to slaughter, it would be very useful in the future to record the age category for each carcass in the FLEKO.

#### *Non-reporting bias*

A large percentage of slaughterhouses operating never reported a single whole carcass condemnation during the 6 years of this study: 63% (486/777), 64% (540/847), and 79% (645/814) for pig, cattle and small ruminant slaughterhouses respectively. Given a condemnation rate between 1-2‰, we would expect slaughterhouses having processed over 1,000 carcasses during the study period to have reported at least one carcass condemnation but not all have. Still, we estimated that 106 pig slaughterhouses processing over 1,000 carcasses over the five years of the study never reported a single carcass condemnation to the FLEKO (the numbers were 35 and 70 slaughterhouses for cattle and small respectively). Caution should therefore be applied when making comparisons between the Swiss data and other carcass condemnation data as a result of potential under-reporting.

### Reasons for carcass condemnation

Overall, between 55 and 59% of condemnation codes were used with a frequency of less than 1% for the three animal types (Table 5). Each type of production animal appears to have a specific profile when it comes to reasons evoked for whole carcass condemnations. “Acute lesions” was the most common cause of carcass condemnation for cattle (both normal and emergency slaughters) and small ruminant (emergency slaughter); while abscesses were the most common cause for pigs (both normal and emergency slaughters). Pronounced weight loss was the most commonly reported cause of condemnations for small ruminant condemnations during normal slaughter. These reasons could constitute valuable syndromic indicators as they are unspecific clinical manifestations of a large range of animal diseases as well potential indicators of animal welfare. Similarly, condemnations for severe injuries or pronounced weight loss could be used, among others, as animal welfare indicators to identify farms on which management practices could be improved.

**Table 5:** Reasons for whole carcass condemnations

	**Pigs**		**Cattle**		**Small ruminants**	
	**N**	**E**	**N**	**E**	**N**	**E**
Anthrax	-	-	-	<1	-	-
Blackleg	-	-	-	<1	-	-
Tuberculosis	<1	-	<1	-	-	-
Salmonellosis	-	-	<1	<1	-	-
TSE	-	-	<1	<1	-	-
Actinobacillosis	-	<1	<1	<1	-	-
Lymphadenitis	<1	-	<1	<1	<1	-
Tetanus	-	-	-	<1	-	<1
Erysipelas	4.8	<1	-	-	-	-
Botulism	<1	-	-	<1	-	-
Listeriosis	-	-	<1	<1	<1	<1
Sarcosporidiosis	<1	<1	3.4	<1	1.3	-
Cysticercosis	<1	-	1.7	<1	1.1	-
SPSTBV	6.5	6.8	10.5	11.6	5.25	9.6
Tumors	1.3	<1	1.4	<1	<1	<1
Abcesses	**35.2**	**36.0**	12.4	6.5	12.4	6.3
Severe injuries	<1	3.0	1.7	6.6	<1	2.8
Pronounced weight loss	2.6	5.8	8.5	8.9	**23.0**	15.4
Acute lesions	22.3	21.6	**23.0**	**14.6**	15.2	**17.8**
Animal arrived dead	3.0	<1	<1	<1	<1	<1
Animal agonising	<1	4.3	<1	4.2	<1	4.4
No pre-mortem control	<1	<1	<1	<1	-	-
Some parts not controlled	-	-	-	<1	<1	<1
Aged <7 days	-	-	<1	<1	-	-
Contains risk material	-	-	-	<1	-	-
Animal not bled	<1	<1	<1	1.3	<1	1.3
Soiled/heat-damaged	2.8	<1	<1	<1	<1	-
Colour of carcass	2.9	3.5	6.5	6.9	7.0	6.1
Smell of carcass	7.7	3.4	6.4	4.1	2.6	3.9
Texture of carcass	2.0	4.6	10.2	9.7	11.4	10.4
Flavour of carcass	<1	<1	<1	<1	<1	1.3
Appearance of carcass	5.1	6.7	8.8	12.6	6.1	11.2
> legal value	<1	<1	1.3	4.6	10.1	7.1
Severe intoxication	-	<1	<1	<1	<1	<1
Forbidden substance	<1	<1	<1	4.1	<1	<1
FPT	<1	-	<1	<1	<1	-

Percentage of total condemnations between 2007–2012 per condemnation code per production animal type during normal (N) and emergency (E) slaughters. The most commonly reported reason for each animal type is denoted in bold.

*TSE*: Transmissible spongiform encephalopathies; *SPSTBV*; symptoms of pyaemia, septicemia, toxemia, bacteremia or viremia; *FPT*: forbidden physical treatment.

A further eight condemnation reasons were never reported between 2007–2012: animal dead at birth, trichinellosis, scrapie, brucellosis, highly contagious epizooties, rabies, equine encephalitis and glanders (later two are horse specific).

## Conclusions

The slaughterhouse data routinely collected by the FSVO have the potential to currently contribute to the monitoring of production animals health (in particular endemic diseases) for the whole of Switzerland. One of its advantages is the simultaneous coverage of cattle, pigs and small ruminants, despite the reporting biases we have highlighted. Secondly, because reporting of whole carcass condemnations is, at least in theory, compulsory, the monitoring of particular health events during meat inspection does not necessitate additional work on the part of the meat inspector and can be performed centrally from the FSVO. Importantly, traceability of each condemnation to its farm of origin (either birth farm or last farm visited before slaughter) is possible through the linking of the animal or batch identity number to the national animal movement database allowing for the epidemiological investigation of potential problem farms.

The numbers of condemnations were significantly linked to the total number of animals slaughtered, highlighting the fact that it will be important in the future to offset the condemnation data by this denominator. However, while the date on which a condemnation is made is available, the denominator is often only communicated at the end of the month so that, in practice, condemnations may currently only be monitored on a monthly basis. Coupled with the lack of timeliness (30–60 days delay between condemnation and notification), this limits the use of the data for early-detection of emerging or re-emerging diseases in the Swiss herd. A first step would be to ask the veterinarians in charge of meat inspection to enter their data at the end of each working day into the database, thereby greatly reducing the time gap between observation of an event by the meat inspector and notification to the database. As for the denominator, in the larger slaughterhouses at least, the daily volume of animals slaughtered is known and often automatically recorded as animals or carcasses are placed on the belt or the scale. By building a direct IT interface between the slaughterhouse IT system and the FLEKO data warehouse managed by Identitas SA, it should be able in the future to perform timely analyses of daily condemnation rates.

## Endnotes

^a^Syndromic surveillance is “the real time (or near real-time) collection, analysis, interpretation and dissemination of health-related data to enable the early identification of the impact (or absence of impact) of potential human or veterinary public-health threats which require effective public health action” (Triple-S definition: http://www.syndromicsurveillance.eu/).

^b^817.190.1 Verordnung des Eidgenössische Departement der Innern (EDI) über die Hygiene beim Schlachten (VHyS) vom 23. November 2005.

## Abbreviations

ARMA: Auto-regressive moving average; FSVO: Federal food safety and veterinary office; GAM: Generalized additive models; RSALL: Restricted substance above legal limit; SFR: Swiss francs; SPSTBV: Symptoms of pyaemia, septicemia, toxemia, bacteremia or viremia.

## Competing interests

The authors declare that they have no competing interests.

## Authors’ contributions

FV & MR conceived and participated in the design of the study. FV performed the statistical analyses and drafted the manuscript. Both authors read and approved the final manuscript.
